# Emerging Strategies against Non-Typhoidal *Salmonella*: From Pathogenesis to Treatment

**DOI:** 10.3390/cimb46070442

**Published:** 2024-07-14

**Authors:** Cristina Mihaela Sima, Elena Roxana Buzilă, Felicia Trofin, Diana Păduraru, Cătălina Luncă, Alexandru Duhaniuc, Olivia Simona Dorneanu, Eduard Vasile Nastase

**Affiliations:** 1 Department of Preventive Medicine and Interdisciplinarity—Microbiology, “Grigore T. Popa” University of Medicine and Pharmacy, 700115 Iasi, Romania; cristina.sima@umfiasi.ro (C.M.S.); elena-roxana.buzila@umfiasi.ro (E.R.B.); felicia.trofin@umfiasi.ro (F.T.); catalina.lunca@umfiasi.ro (C.L.); alexandru.duhaniuc@umfiasi.ro (A.D.); 2 Clinical Hospital of Infectious Diseases “Sf. Parascheva”, 700116 Iasi, Romania; eduard-vasile.nastase@umfiasi.ro; 3 Iasi Regional Center for Public Health, National Institute of Public Health, 700465 Iasi, Romania; 4 “Dr. C.I. Parhon” Clinical Hospital, 700503 Iasi, Romania; diana_paduraru@email.umfiasi.ro; 5 “Sf. Maria” Children Emergency Hospital, 700309 Iasi, Romania; 6 Department of Internal Medicine II—Infectious Diseases, “Grigore T. Popa” University of Medicine and Pharmacy, 700115 Iasi, Romania

**Keywords:** non-typhoidal *Salmonella*, iNTS, pathogenesis, diagnostic methods, antimicrobial resistance, probiotics, vaccination

## Abstract

Even with the intensive efforts by public health programs to control and prevent it, non-typhoidal *Salmonella* (NTS) infection remains an important public health challenge. It is responsible for approximately 150 million illnesses and 60,000 deaths worldwide annually. NTS infection poses significant risks with high rates of morbidity and mortality, leading to potential short- and long-term complications. There is growing concern among health authorities about the increasing incidence of antimicrobial resistance, with multidrug resistance totaling 22.6% in Europe, highlighting an urgent need for new therapeutic approaches. Our review aims to provide a comprehensive overview of NTS infection. We outline the molecular mechanisms involved in the pathogenesis of NTS infection, as well as the events leading to invasive NTS infection and the subsequent complications associated with it. Given the widespread implications of antimicrobial resistance, our review also presents the global landscape of resistance, including multidrug resistance, and delve into the underlying mechanisms driving this resistance. The rising rates of antibiotic resistance frequently lead to treatment failures, emphasizing the importance of investigating alternative therapeutic options. Therefore, in this review we also explore potential alternative therapies that could offer promising approaches to treating NTS infections.

## 1. Introduction

Foodborne pathogens pose significant threats to food safety and public health as these pathogens and their toxins can result in various human diseases when consuming contaminated animal products or water [1]. According to the World Health Organization (WHO), *Salmonella* spp. rank among the top 31 pathogens with the greatest potential to cause intestinal or systemic diseases in humans [2,3]. It is responsible for approximately 150 million illnesses and 60,000 deaths worldwide annually [4]. Annually, in the United States, salmonellosis is estimated to result in over 1.2 million illnesses, 23,000 hospitalizations, and 450 deaths [5]. In 2022, European countries reported 66,721 cases of salmonellosis, with 65,967 classified as laboratory confirmed. Hospitalization status was documented for 29,712 cases, of which 39.3% required hospitalization. There were 81 reported deaths, resulting in a case fatality rate of 0.22% [6]. In Europe, *Salmonella* ranked as the second most frequently reported foodborne pathogen after campylobacteriosis, playing a significant role in foodborne outbreaks across both the European Union (EU) member states and non-member countries [7]. Additionally, *Salmonella* is the third leading cause of death from foodborne diseases, after Enteropathogenic *E. coli* and norovirus [2,8].

*Salmonella* is a facultatively anaerobic Gram-negative bacterium that belongs to the *Enterobacteriaceae* family. It can survive under a wide range of conditions, being capable of growing at temperatures between 8 °C and 45 °C and in environments with a pH ranging from 4.0 to 9.5. *Salmonella* spreads by the fecal–oral route, commonly through contaminated food, water, and inadequate sanitation [9,10]. According to the White–Kauffmann–Le Minor scheme, around 2659 *Salmonella* serovars have been identified [11]. The species can be broadly categorized into human-restricted typhoidal *Salmonella* and zoonotic non-typhoidal *Salmonella* (NTS) [7]. In our review, we address solely NTS and its implications in pathology.

The United States and European Food Safety Authority(EFSA) recognize *S*. Enteritidis and *S*. Typhimurium as the two most common serotypes responsible for human NTS infections. However, other frequent serotypes, such as Newport, Javiana, Infantis, and monophasic Typhimurium 4,[5],12:i:-, have also been shown to cause disease [12,13]. Animals are the main source of NTS, with animal-based foods being the primary route of transmission to humans. A variety of domestic and wild animals can host *Salmonella* and serve as reservoirs, including poultry, swine, cattle, wild birds, rodents, pets, and exotic animals [14]. The prevalence in different food matrices varies across countries and regions, shaped by cultural and food production practices, geographic location, and economic factors [8]. Pigs are a major source of NTS infections in humans. In many countries, including most of Europe and the United States, they are considered a leading source of salmonellosis. This is because pigs often carry and spread the pathogen asymptomatically throughout the production chain. Consequently, pigs and pork meat are the primary sources of the Typhimurium serovar [12,15]. Poultry can acquire various *Salmonella* serovars, often without displaying symptoms, and so they become disseminators of *Salmonella* through both horizontal and vertical routes [16]. Serovar Enteritidis is linked to the consumption of poultry and eggs [9]. In Europe, 57.2% of *S.* Enteritidis isolates were found in broiler chickens and their meat, while 37.1% were found in laying hens and eggs [13]. Other serovars like Anatum and Weltevreden were commonly detected in beef and seafood, respectively [2]. Weltevreden serovar is recognized as a significant pathogen in public health, particularly in the coastal regions of China [17].

NTS infection occurs when bacteria bypass the gastric acid barrier and invade intestinal epithelial cells, initiating inflammation. Subsequently, this leads to diarrhea, ulceration, and the destruction of mucosal cells. If the infection persists and spreads within the intestines, it can result in an invasive NTS (iNTS) systemic infection, particularly in individuals with weakened immune systems and newborns [18,19]. iNTS infections have the highest morbidity and mortality in developing countries, contributing to a worldwide burden of 3.4 million cases and over 680,000 deaths [19]. Although NTS infection represents a significant public health issue, its pathogenesis is not yet fully understood [20]. In this review, we aim to provide a comprehensive overview of the known molecular mechanisms involved in the pathogenesis of NTS infection, as well as the events leading to iNTS infection and the subsequent complications that appear in correlation to it. As the incidence, mortality, and morbidity of iNTS infections are increasing in developing countries [19], there is a real need for tests that offer a rapid and accurate diagnosis, but are also affordable. Thus, in our review, we present the current diagnostic methods and what the future holds for us in this area.

The treatment approach for NTS infections primarily involves supportive care, such as oral rehydration therapy. Antibiotic treatment is typically not advised for the majority of patients since it does not reduce the duration of illness and might extend the period of bacterial shedding. Antibiotic therapy should be considered for patients showing signs of invasive disease or those with a heightened risk of developing it [4]. However, abusive antibiotic administration has led to the emergence of *Salmonella* strains that are becoming more resistant or even multi-resistant to conventional antimicrobials [14]. A matter of concern is the worldwide rise in multidrug resistance (MDR) among NTS. Research indicates that individuals with antimicrobial-resistant NTS infections are at a higher risk of developing bloodstream infections and requiring hospitalization [21]. Considering the numerous implications of antimicrobial resistance, our review addresses the global situation regarding both antimicrobial and multidrug resistance, alongside exploring the underlying mechanisms of this resistance.

Last but not least, the increasing rate of antibiotic resistance often leads to therapeutic failure. Therefore, it is absolutely necessary to turn to other therapeutic alternatives. One of the alternative therapeutic options is probiotics. Probiotics are defined as living microbes that, when administered correctly, provide health benefits to the host [22]. Our review outlines the present status of probiotic utilization in the treatment of NTS infection and explore the available viable alternatives. With the escalating number of iNTS infections, associated with high morbidity and mortality [23], and the growing incidence of multidrug resistance [24], disease prevention with vaccines remains the optimal strategy. Currently, there are no approved vaccines against NTS [25]; however, several options are in various stages of development. In our review, we discuss the existing options for NTS vaccination and provide an overview of their current status.

The literature review for this article included an extensive search across various scientific databases, such as PubMed, Embase, and Cochrane. Studies were identified using a combination of search terms including “*Salmonella*”, “pathogenesis”, “diagnosis” and “treatment” with Boolean operators (AND, OR) applied to refine search results. The initial search resulted in 8456 articles, which were screened based on their titles and abstracts to exclude irrelevant studies. After screening the titles and reviewing the abstracts, full-text articles of potentially relevant studies were retrieved and further assessed. A thorough examination of the full texts resulted in the exclusion of additional articles due to relevance issues, methodological incongruity, scope misalignment, quality and credibility concerns, or language barriers. Furthermore, supplementary references meeting the defined criteria were identified through a manual search of the reference lists of retrieved articles. After completing this process, our review included 181 studies.

## 2. Pathogenesis

**Figure 1:**
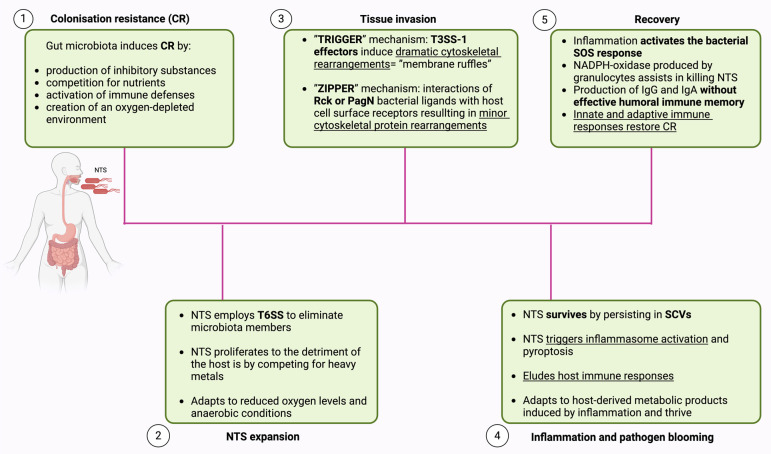
Pathogenesis of NTS infection. Image created with BioRender.com using information from references [32,33,34,35,36,37,38]. NTS: non-typhoidal *Salmonella*; T6SS: type 6 secretion system; T3SS-1: type 3 secretion system encoded by SPI-1; SCVs: *Salmonella*-containing vacuoles.

The symptoms of NTS infection are usually self-limiting and typically last for about a week [26]. The incubation period ranges from 6 h to 6 days after exposure, with the infection usually lasting 4 to 7 days. Bacteria may be shed in feces for a month or longer [27]. The most common symptoms include gastroenteritis, with clinical signs such as nausea, vomiting, headache, abdominal pain, diarrhea, and muscle pain. Prolonged fluid loss can result in dehydration, potentially fatal for newborns and older adults if fluid balance is not maintained [28]. The strains of *Salmonella* that are known to be invasive, iNTS, are particularly effective at causing systemic disease. They are especially concerning for adults with HIV and for children in sub-Saharan Africa who are diagnosed with HIV infection or malaria, or who are malnourished [29,30]. A number of factors related both to the pathogen and the host influence the pathogenesis of NTS infection. Next, we describe the pathogenesis of NTS infection (Figure 1) and introduce new insights into its molecular mechanisms. For simplicity, we follow the five phases proposed by Stecher in 2021 [31].

### 2.1. Colonization Resistance (CR)

Upon ingestion, NTS encounters a complex environment influenced by dietary factors, immune defenses, and the microbial community in the gastrointestinal tract. A considerable number of individuals who are exposed to the infection do not experience symptomatic illness, indicating that their bodies effectively resist the infection through a phenomenon known as CR [31]. The molecular mechanisms underlying CR are highly varied. Certain strains of microbiota produce inhibitory substances like bacteriocins, lantibiotics, and colicins, which effectively suppress the growth of invading pathogens. Another mechanism for CR is the competition for binding sites on the mucus surface or epithelium. Additionally, the resident microbiota creates an oxygen-depleted environment, which hampers the growth of facultative anaerobic pathogens like NTS. Moreover, microbiota are thought to compete for nutrients, potentially inhibiting pathogen growth. Lastly, the prior activation of immune defenses by the microbiota may impact infection progression after exposure to pathogens [32,33].

Research has revealed that *E. coli* offers resistance to NTS infection by competing for fumarate as an electron acceptor and reducing the availability of galactitol that would otherwise support the growth of the incoming pathogen [39,40]. Another way in which microbiota hinder the proliferation of invading pathogens is through the production of short-chain fatty acids (SCFAs) by species like *Clostridia* and *Bacteroidia*. SCFAs limits the range of successful metabolic strategies available to NTS and exhibit antimicrobial properties against it [31,41]. A recent study unveiled how an oxygen-depleted environment contributes to CR. It seems that reduced oxygen levels significantly diminish NTS adhesion. This change in adhesion behavior correlates with decreased pathogen motility and a notable reduction in biofilm formation, attributed to lower flagellin expression [42].

Studies have demonstrated that the ability of microbiota bacteria to establish CR depends on the microbial context [40]. This theory is further supported by a 2023 study by Spragge et al., which examined the CR offered by human gut symbionts against two significant bacterial pathogens, *Klebsiella pneumoniae* and *Salmonella enterica* serovar Typhimurium. The findings revealed that even the most effective species offered limited protection against the pathogens when acting alone. However, when the species were combined into diverse communities of up to 50 species, pathogen growth was significantly inhibited. The key to effective CR was the overlap in nutrient-utilization profiles between the microbial community and the pathogen [43].

The newest discovery about CR implies the role of nociceptor sensory neurons (responsible for detecting pain from harmful stimuli). These neurons were found to protect the host from NTS colonization, invasion, and dissemination. They do so by regulating the density of M-cells in Peyer’s patches, thereby inhibiting the entry of NTS. Additionally, nociceptor neurons help maintain the levels of segmented filamentous bacteria in the gut microbial community, which plays a crucial role in mediating resistance to NTS [44].

### 2.2. NTS Expansion

When NTS reaches the large intestine, it can begin to proliferate if conditions are favorable and CR is not an obstacle. Having survived the acidic pH of the stomach, exposure to bile acids, antimicrobial peptides, and adaptation to reduced oxygen levels and anaerobic conditions, NTS can proliferate to densities sufficient to cause disease [32]. In fact, NTS exhibits greater inherent resistance to bile salts when compared to gut commensals. The contemporary diet, often high in fat, triggers an increased release of bile salts into the gut following fat ingestion. This environment can promote the colonization of pathogens over commensal bacteria [45].

Virulence genes responsible for NTS pathogenicity are located in *Salmonella* pathogenicity islands (SPIs), which are represented by large clusters of genes within the *Salmonella* genome. These SPIs encode membrane-associated secretion systems that produce various proteins involved in NTS virulence [46]. To outcompete bacterial competitors, NTS employs the type VI secretion system (T6SS) to eliminate microbiota members through contact-dependent mechanisms [34]. The T6SS is a nanomachine that functions through dynamic cycles of assembly, contraction, and disassembly. It facilitates the direct delivery of toxins into eukaryotic or prokaryotic cells [47]. To avoid self-intoxication, these toxin proteins are generated together with specific cognate immunity proteins that protects the donor cell [48,49]. Nevertheless, a recent study has demonstrated the possibility of contact-independent killing by a T6SS. It secretes an effector called Tce1, a small protein that functions as a Ca^2+^ and Mg^2+^ dependent nuclease, effectively killing other bacteria [50]. Another mechanism through which NTS proliferates to the detriment of the host is by competing for heavy metals. Heavy metals are essential for the survival of all living organisms, including bacteria. During infection, NTS produces siderophores, such as enterochelin (enterobactin) and salmochelins (SX, S1, S2, and S4), to sequester Fe3+ from the extracellular medium. The advantage of salmochelins is that they are not bound by lipocalin-2, a siderophore-capturing protein, unlike enterobactin, thereby conferring NTS an advantage against the host [51].

Once NTS overcomes initial adversities, it begins to multiply rapidly. Studies show that NTS reaches high densities (approximately 10^9^ CFU/g stool) between days 1 and 10 of infection, which seems essential to ensure that a sufficient number of bacteria can invade the gut epithelium [52].

### 2.3. Tissue Invasion

To initiate invasion, NTS first attaches to epithelial cells using fimbrial and non-fimbrial adhesins. Key fimbrial adhesins include type 1 fimbriae (Fim), plasmid-encoded fimbriae (Pef), long polar fimbriae (Lpf), and thin aggregative fimbriae (Agf). Non-fimbrial adhesins like MisL and the Type 1 Secretion System (T1SS), encoded by SPI-4, also play crucial roles [48]. T1SS produces SiiE, a non-fimbrial adhesin, enabling NTS to attach to polarized epithelial cell microvilli via its N-terminal moiety. SiiE is not required for adhesion in non-polarized cells [53].

Until recently, *Salmonella* invasion of eukaryotic cells was thought to rely solely on the type III secretion system (T3SS) [54], which acts as a molecular syringe delivering effector proteins into host cell cytosols [55]. T3SS-1, encoded by SPI-1 primarily facilitates cell invasion, while T3SS-2, encoded by SPI-2, supports *Salmonella* survival within host cells [54]. However, new evidence reveals that NTS can cause infection independently of T3SS-1. Two outer membrane proteins, Rck and PagN, are now recognized as key *Salmonella* invasins [56]. Essentially, *Salmonella* employs both “Trigger” and “Zipper” mechanisms to invade host cells [35].

In the ”Trigger” mechanism, the effectors of T3SS-1 engage with the host actin cytoskeleton, initiating the formation of membrane ruffles (dramatic cytoskeletal rearrangements). These ruffles engulf NTS into a confined intracellular space, exceeding the size needed for a single bacterium and enabling the simultaneous internalization of multiple bacteria—a process known as cooperative invasion [57]. The main effectors involved in T3SS-1-mediated uptake are SopB, SopE, and SopE2. They enhance actin polymerization indirectly by boosting the function of nucleation promoting factors like WAVE and WASH [58,59]. In contrast, SipA and SipC act directly on actin, possessing nucleation and bundling capabilities that promote membrane ruffles and aid invasion [60].

In the “Zipper” mechanism, PagN and Rck activate cytoskeletal regulators via the phosphatidylinositol 3-kinase (PI3K) signaling pathway. This activation occurs due to their interaction with host receptors such as EGFR or heparin-binding proteoglycans, leading to minor rearrangements of cytoskeletal proteins [61]. Despite this, Rosselin et al. demonstrated significant invasion of fibroblasts, epithelial, and endothelial cells by an NTS strain even in the absence of Rck, PagN, and T3SS-1 [62]. This suggests potential alternative invasion pathways, opening new avenues for identifying novel invasion factors.

### 2.4. Inflammation and Pathogen Blooming

The invasion into intestinal epithelial cells is a central step in the infection cycle and is associated with gut inflammation. In order to induce inflammation, NTS must survive within host cells [63]. NTS survives by persisting in vesicles called Salmonella-containing vacuoles (SCVs). It employs various strategies, primarily using T3SS-2 to dampen the host’s immune response. T3SS-2 enhances intracellular survival by inhibiting autophagy via multiple effector proteins [36]. SopF inhibits autophagy by ADP-ribosylating ATP6V0C, a component of the vacuolar (H+)-ATPase (V-ATPase) [64]. SseL deubiquitinates SCVs, reducing autophagy markers like p62 and LC3 [65]. SseF and SseG disrupt Rab1A signaling, preventing autophagosome formation [66]. Additionally, SpvC inhibits autophagosome formation through its phosphothreonine lyase activity [37], and SpvB, from the pSLT plasmid, hinders autophagy by depolymerizing the actin cytoskeleton [67]. SseK1, SseK2, and SseK3 inhibit the TNF-α-triggered NF-κB pathway, suppressing the immune response [68]. Anti-virulence agent A (AvrA), another T3SS-2 effector, inhibits NF-κB via acetylation of MAPK kinase 4 and MAPK kinase 7, thereby blocking the JNK signaling pathway [69].

Upon infection, NTS triggers inflammasome activation and pyroptosis in epithelial and immune cells. This starts with the NAIP/NLRC4 inflammasome, leading to IL-18 release and IFNγ production [70]. The activation of the NAIP/NLRC4 inflammasome is induced by bacterial protein ligands like flagellin and the T3SS needle protein PrgI [71].

Macrophages are crucial for defending against NTS by phagocytosing the pathogen after it breaches the epithelial layer [55]. They exist as M1 and M2 macrophages. M1 macrophages are activated by microbial pathogen-associated molecular patterns (PAMPs) and cytokines, resulting in killing of the pathogens. M2 macrophages are activated by Th2 cytokines, aiding in tissue repair and defense against parasites but can also promote fibrosis and tumors [72]. NTS uses the T3SS-2 effector, SteE, to shift macrophages from M1 to M2, enhancing its survival [73]. A recent study showed that lactate produced by macrophages induces T3SS-2, enhancing the translocation of SteE and promoting M2 polarization [74].

Dendritic cells’ antigen presentation to CD4+ T cells is vital in fighting infections. T3SS-2 effector, SteD, interacts with mature MHC-II, causing its degradation and directly impeding antigen presentation. SteD also reduces surface CD86 levels, crucial for full T-cell activation, thus inhibiting T-cell response during NTS infection [75]. Moreover, SteD induces ubiquitination and degradation of CD97, disrupting dendritic cell-T cell interactions and independently reducing T-cell activation [76].

NTS can cause severe invasive bacteremia, known as iNTS disease. Susceptibility to iNTS infection is intricately tied to immunological mechanisms involving various cytokines and lymphocytes. Upon intestinal penetration, macrophages recognize the bacteria, activating T cells and NK cells through IL-12 and IL-23. These activated cells release interferon-γ, which enhances macrophage activity via STAT1, aiding pathogen clearance. Inadequate cytokine expression or lymphocyte depletion can impair intracellular killing, contributing to severe infections in immunocompromised patients [77]. Studies on the NLRC4 inflammasome in NTS infection have mostly focused on gastrointestinal models, the primary route of infection. In contrast, the impact of inflammasome activation and pyroptosis in iNTS infection remains underexplored. Caspase-1 is critical for NTS’s systemic spread. Disruption of the inflammasome in iNTS infection has been found to have both protective and detrimental effects [78]. Interestingly, a study revealed that deletion of ROD21 SPI enhances NTS’s invasion of host cells, which is crucial for its subsequent spread from the digestive tract to internal organs [79]. Macrophages also play a critical role in NTS’s systemic spread. NTS induces apoptosis in the infected macrophages facilitated by SPI-1 effectors. Additionally, inflammasome-triggered pyroptosis leads to cell death, aiding bacterial release and further infection [80]. NTS can also exploit dendritic cells as “Trojan horses” to enhance their spread, a strategy common among diverse pathogens [81].

Therefore, NTS can survive even in the hostile environment generated by inflammation. It replicates by utilizing host-derived metabolic products induced by inflammation, such as thiosulfate, tetrathionate, ethanolamine, and nitrate [38]. Inflammation prompts phagocytes to produce superoxide anion radicals, which oxidize hydrogen sulfide into thiosulfate and tetrathionate [82]. Simultaneously, deceased intestinal cells release ethanolamine from their membranes. *Salmonella* can utilize ethanolamine as a carbon source and tetrathionate for anaerobic respiration. Furthermore, host-generated superoxide anion radicals react with inflammation-induced nitric oxide to produce nitrate, aiding *Salmonella’s* intestinal growth as an electron acceptor [83]. Furthermore, *Salmonella* can thrive by utilizing metabolites generated by other intestinal microorganisms, such as lactic acid and propionic acid [84,85].

In summary, NTS induces inflammation by inflammasome activation and pyroptosis and is capable of using various strategies to evade host immune responses. The roles of many *Salmonella* proteins and their interactions with host cells are still unclear, leaving much to explore about *Salmonella’s* pathogenicity.

### 2.5. Recovery

Resolution of NTS infection involves both innate and adaptive immune mechanisms, as well as the re-establishment of CR [32]. The first line of defense against NTS infections is innate immunity, relying on the complement system and phagocytosis by macrophages, neutrophils, and dendritic cells. IFNγ and antibodies from adaptive immunity also enhance this response [86]. Essentially, inflammation activates the bacterial SOS response, helping to decrease pathogen levels in the gut lumen, while NADPH-oxidase produced by infiltrating granulocytes assists in killing the pathogen [87]. Finally, the infection prompts the production of IgG and IgA antibodies as part of the immune response [32]. Regarding IgG, *Salmonella* appears to reduce the longevity of IgG-producing plasma cells in the bone marrow (the main source of serum IgG) by secreting the SiiE effector via T3SS. This action hinders the development of an effective humoral immune memory [88]. On the other hand, high-affinity IgA is secreted into the gut lumen, where it binds to the pathogen’s surface, preventing its access to the mucosal surface and speeding up its clearance from the gut lumen [89]. Another study proposes that protection is achieved through a mechanism called “enchained growth.” In this process, IgA coats the bacteria, causing the gut luminal pathogens to form large monoclonal clumps. These clumps prevent tissue invasion, reduce plasmid transfer (which requires contact between donor and recipient bacterial clones), and accelerate pathogen elimination from the gut [90].

Under certain conditions, the immune system may fail to completely eradicate the bacteria, resulting in a persistent NTS infection. Persisters are in a dormant state of infection that exhibits tolerance to drug treatment and can eventually reactivate, resuming replication [91]. This state of persistent infections is associated with multiple complications that we address in the next section. Regarding the development of this prolonged infection, it is determined by the interplay of conflicting signals of immune activation and suppression. For instance, the activity of regulatory T cells during the initial phases of infection contributes to the persistence of NTS infection [92]. Dendritic cells and macrophages serve as crucial reservoirs for bacteria, facilitating their long-term survival as NTS, and can undermine the host immune response and reprogram the cells they inhabit [93]. Finally, the latest discovery reveals that, besides antigen-presenting cells, NTS can establish persistent infection within epithelial cells by entering a dormant state. Luk et al. discovered that NTS can remain dormant within the vesicular compartment, distinct from SCVs, where it can still express SPI-2 virulence factors, unlike in macrophages [94].

## 3. Complications Associated with NTS Infection

**Figure 2:**
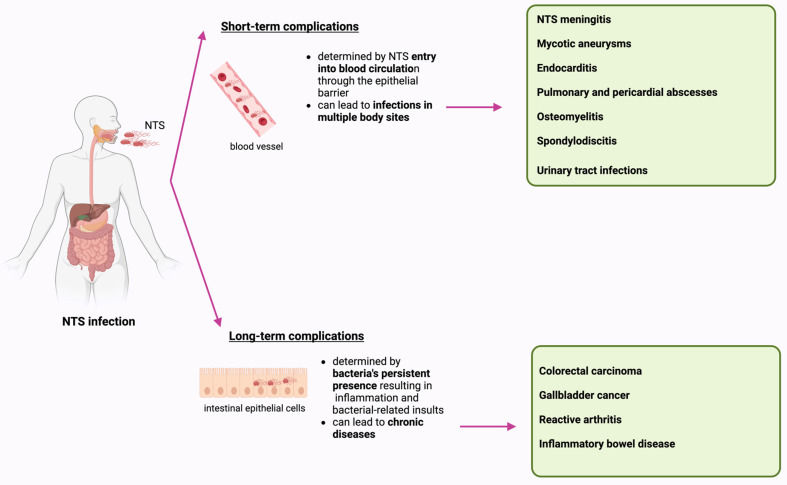
Complications associated with NTS infection. Image created with BioRender.com. NTS: non-typhoidal *Salmonella*.

NTS infections can result in both immediate and lasting complications, highlighting the diverse impact on affected individuals. Short-term complications arise from NTS spreading through the blood, causing infections at multiple body sites, whereas long-term complications are linked to NTS persistence in the host, leading to chronic diseases such as neoplasms and autoimmune disorders (Figure 2) [95,96].

### 3.1. Short-Term Complications

When NTS breaches the epithelial barrier and causes bacteremia, it can lead to infections in multiple body sites. The most critical forms include endovascular and central nervous system infections, where definitive debridement is often not feasible. Other forms include endocarditis, musculoskeletal infections, visceral infections (involving organs like the spleen, liver, heart, lungs, or pleura), and urinary tract infections [95].

NTS meningitis can occur in infants, especially those under 3 months and immunocompromised children [97]. While rare in healthy children past infancy, documented cases exist in South Korea, linked with significant morbidity and lasting neurological sequelae. Complications may involve seizures, abscesses, hydrocephalus, and subdural empyema, potentially causing severe developmental delays and motor impairments [98].

Atherosclerosis and prior vascular interventions predispose individuals to vascular complications from NTS infection [99]. Mycotic aneurysms are rare but severe complications of iNTS infection, characterized by localized arterial wall dilatations due to infective processes causing vessel wall destruction. They have a higher rupture incidence and mortality rate compared to atherosclerotic aneurysms. Ansari et al. reported a case of mycotic aortitis in a patient with multiple comorbidities and rheumatoid arthritis under immunosuppressant therapy, highlighting the importance of early diagnosis and treatment for survival [100].

Endocarditis is a serious complication of NTS bacteremia, especially in patients with prosthetic valves [95]. This invasive infection is highly destructive with a complicated course and a mortality rate exceeding 45%. Diagnostic challenges arise due to the lack of unified criteria, necessitating a high index of suspicion, particularly in patients with pre-existing heart conditions [101]. Although rare, cases of *Salmonella* endocarditis on native valves have been reported [102]. Therefore, clinicians should consider cardiovascular imaging in patients with persistent fever and positive blood cultures to promptly detect and treat potentially fatal *Salmonella* endocarditis.

*Salmonella*-induced pulmonary and pericardial abscesses have been documented in patients with a subacute cough. These rare pleuro-pulmonary manifestations primarily occur in individuals with pre-existing lung conditions [103].

*Salmonella* osteomyelitis is more common in children than adults, often linked to contact with pet reptiles, even without prior diarrhea. Children with hemoglobinopathies, like sickle cell disease, are particularly vulnerable due to repeated vaso-occlusive crises that damage the gut and bones, facilitating bacterial entry into the bloodstream and subsequent bone colonization [104]. In adults, infections typically affect the axial skeleton, especially the lumbar spine, whereas in prepubescent children, infection is more likely to occur at the epiphysis of long bones [105]. Another complication associated with NTS is spondylodiscitis, a rare condition. Unlike osteomyelitis, which is more common in children with haemoglobinopathies, spondylodiscitis can occur in healthy, immunocompetent children [106].

Finally, NTS has been shown to potentially cause urinary tract infections (UTIs). Predisposing factors include chronic diseases like diabetes and immunodeficiency, as well as genitourinary tract abnormalities such as nephrolithiasis, chronic pyelonephritis, and fistulas (urethrorectal or retrovesical). Importantly, NTS UTIs can also affect healthy, immunocompetent individuals [107].

### 3.2. Long-Term Complications

NTS weakens the host immune system, enabling longer survival within the host. The bacteria’s persistent presence leads to inflammation and bacterial-related insults, increasing the host’s susceptibility to debilitating diseases. NTS persistence is linked to chronic diseases like colorectal and gallbladder carcinoma, as well as autoimmune disorders [96].

#### 3.2.1. Colorectal Carcinoma

Viral infections are widely acknowledged as contributing to cancer development, whereas bacterial infections are rarely considered as causal factors. However, Mughini-Gras et al. found that severe NTS infection raises the risk of colon cancer in the ascending/transverse regions of the colon [108], a view supported by other studies [109]. Repeated infections also heighten the risk of colorectal carcinoma and speed up tumor growth [110].

Next, we aim to explore how NTS is involved in the development of colorectal cancer. T3SS secretes over 40 effector proteins into host cells [111], with SopB (also called SigD) and AvrA identified as key effectors in colon cancer development [112]. SopB activates the Akt pathway to prevent cell apoptosis, critical in *Salmonella*-induced colon cancer [96]. AvrA suppresses NF-κB and JNK pathways and inhibits apoptosis. AvrA inhibits apoptosis also by reducing Beclin-1, a critical regulator of autophagy, via the JNK pathway [113]. AvrA functions as both a deubiquitinase and acetyltransferase. Its acetyltransferase activity acetylates key amino acids in MAP kinase, blocking its phosphorylation and inhibiting JNK and NF-κB pathways. This activation promotes STAT3 signaling, contributing to colitis-associated colon cancer [114]. AvrA could thus serve as a biomarker for early colon cancer detection, guiding the development of new therapies. It is important to note that *Salmonella*-induced changes in host signaling pathways are only part of the story. NTS can also alter the gut microbiota during infection [115], which may contribute to colorectal cancer development. Further studies are needed to understand how *Salmonella* disrupts the human colon microbiome, impacting metabolites and inflammation, and increasing the risk of tumorigenesis and chronic diseases.

#### 3.2.2. Gallbladder Cancer (GBC)

GBC is a prevalent malignancy in the hepatobiliary system, ranking sixth among gastrointestinal cancers. Chronic *Salmonella enterica* infection is a major risk factor associated with GBC [96]. While Typhi strains of *Salmonella* are primarily linked to GBC, recent exome sequencing of GBC patient samples has also identified NTS DNA [116]. Effector proteins like SipA, SopB, SopE, SopE2, and SptP play crucial roles in the pathogenesis of GBC by aiding in cell invasion and activating MAPK and AKT pathways to promote cell transformation [117]. However, genetic mutations like TP53, ERBB2, KRAS, and c-Myc are reported to promote tumorigenesis in gallbladder carcinoma [118]. Gallstones are a significant factor in GBC development, creating an inflammatory environment and promoting *Salmonella* biofilm formation [119]. Bile’s antimicrobial properties stimulate exopolysaccharide production with O-antigens, facilitating *Salmonella* biofilms on gallstones. Bile and gallstones provide nutrients and modulate protein expression, supporting biofilm growth [120]. In the long term, persistent infection may continuously harm genetically susceptible individuals, potentially leading to cellular transformation. Thus, *Salmonella* infection might represent one stage in the multistep process of cancer development.

#### 3.2.3. Reactive Arthritis (ReA)

ReA is a condition characterized by joint inflammation typically appearing weeks after gastrointestinal or genitourinary infections, commonly triggered by enteric bacteria such as *Salmonella*, *Shigella*, *Yersinia*, *Campylobacter*, and *Chlamydia* [121]. Approximately 3.9% of NTS-infected patients may develop ReA according to a recent meta-analysis [122]. Factors influencing ReA susceptibility include age, sex, bacterial load, and HLA-B27 presence. Recent research found a higher ReA incidence linked to HLA-B27 in *Salmonella* infected patients compared to other enteric infections, suggesting HLA-B27 screening for early diagnosis and treatment [123]. Furthermore, HLA-B27 was shown to enhance *Salmonella* replication, indicating a role in regulating *Salmonella* gene expression and growth during infection. This process occurs due to HLA-B27’s tendency to misfold which triggers the unfolded protein response (UPR), favoring *Salmonella* persistence in the gut. This link highlights a pathway in ReA pathogenesis, connecting HLA-B27’s properties with the *Salmonella* life cycle. Targeting the UPR pathway in future therapies may prevent *Salmonella*-induced ReA [124].

The exact mechanism of ReA pathogenesis remains unclear, but it is thought that microbial antigens may inappropriately interact with self-proteins, triggering a prolonged immune response [125]. Another theory, the “arthritogenic peptide” hypothesis, proposes that HLA-B27 activation by CD8+ T cells responding to bacterial peptides could initiate ReA. Cross-reaction of these activated cells with self-peptides may lead to autoimmune tissue damage and inflammation in joints and other tissues. In other words, *Salmonella* has been identified as a bacterium that could trigger HLA-B27-restricted CD8+ T cell responses [126]. The latest theory on ReA pathogenesis focuses on curli, amyloid fibers on enteric bacterial cells that activate pattern recognition receptors (PRRs), triggering inflammatory responses. *Salmonella* produces curli during natural oral infection in mice, particularly in the cecum and colon. Curli production correlates with elevated anti-dsDNA autoantibodies and joint inflammation in infected mice, suggesting curli’s presence in the gut plays a critical role in ReA development [127].

#### 3.2.4. Inflammatory Bowel Disease (IBD)

IBD is a chronic condition characterized by recurrent inflammation of the gastrointestinal tract. Its cause is multifactorial, involving genetic susceptibility, immune system dysregulation, microbial factors, and environmental triggers [128]. Infection with NTS is recognized as a risk factor for IBD development [129], with recurrent infections potentially triggering IBD relapses [130]. NTS initiates the onset of IBD by disrupting the mucosal barrier, altering intestinal microbiota, and affecting immune responses [131].

Recent findings show that *Salmonella* produces membrane vesicles (MVs), crucial in IBD development. These nano-sized, lipid-bilayered structures form through outer membrane blebbing and cell lysis [132]. Once generated, MVs have the ability to disrupt the integrity of the intestinal epithelial barrier, allowing bacteria and products to enter the lamina propria, triggering the immune system and chronic inflammation. Pro-inflammatory molecules like lipopolysaccharides (LPS) and flagellin in MVs activate Toll-like receptors (TLRs) on host cells. This initiates inflammatory pathways and cytokine production, which contribute to the inflammatory condition characteristic of IBD [128]. Another mechanism by which NTS contributes to the pathogenesis of IBD is by reducing granule production and lysozyme secretion by Paneth cells via p38/MAPK activation. Additionally, *Salmonella* induces hyperplasia of Paneth cells via activation of the Wnt pathway. Changes in the proliferation and differentiation of Paneth cells will ultimately alter lysozyme secretion, leading to consequent changes in the microbiota. These changes create a new niche for infection, potentially leading to disease onset with different phenotypes [131].

Several genetic anomalies increase susceptibility to IBD. Mutations in genes associated with IBD, such as XBP1, IL-10, and ATG16L1, predispose individuals to intestinal inflammation and immune disorders. Combined with NTS infection, these genetic changes may trigger early onset of IBD [96]. Most studies on ATG16L1 mutations focus on homozygous animals, although most humans are heterozygous for this allele. A recent study found that heterozygosity for ATG16L1 protects mice from NTS infection. In vivo, a single copy of ATG16L1 is enough to increase inflammatory cytokine production, which is essential for protection against *Salmonella* [133].

In summary, the virulence factors of *Salmonella* that cause inflammation and disrupt epithelial junctions, combined with individual susceptibility, contribute to the pathogenesis of IBD.

## 4. Diagnostic Methods

**Figure 3:**
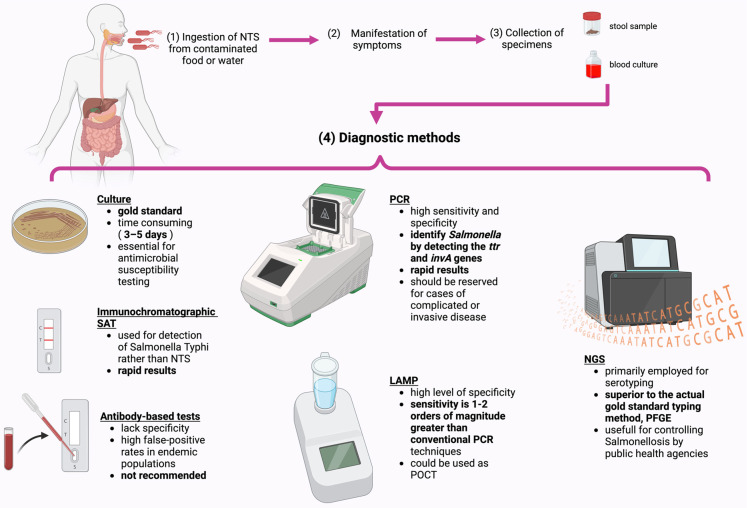
Diagnostic methods for NTS infection. Image created with BioRender.com. SAT: *Salmonella* Antigen Test; LAMP: loop-mediated isothermal amplification; POCT: point-of-care test; NGS: next-generation sequencing; PFGE: pulsed-field gel electrophoresis.

Advances in molecular biology have led to the development of rapid diagnostic tests, which are increasingly used by laboratories for their speed and efficiency. Nonetheless, traditional methods remain valuable, offering accessible and cost-effective options for disease diagnosis (Figure 3). These conventional techniques often complement rapid tests, providing confirmatory results and maintaining their relevance in modern diagnostics [134].

For NTS infections, culture, although time consuming (often taking 3–5 days), is the is still the gold standard in diagnosis, with approximately 90% of isolates derived from routine stool cultures. When iNTS is suspected, a blood culture can be used for diagnosis. Culture is also essential for antimicrobial susceptibility testing [4]. Stool samples are cultured on selective media and enrichment broth, and suspicious *Salmonella* colonies are identified using either automated or traditional biochemical methods. Presumptive isolates are confirmed by serotyping with O and H antisera [134]. Currently, pulsed-field gel electrophoresis (PFGE) represents the gold standard for Salmonella subtyping [135].

In today’s fast-paced world and with the increased number of severe cases, rapid diagnostic tests can significantly reduce diagnosis time, allowing for prompt initiation of etiological treatment. As a result, several rapid tests for diagnosing NTS infection have been developed.

Multiple rapid antigen detection tests, such as the immunochromatographic *Salmonella* Antigen Test (SAT), have been developed. However, their accuracy has mainly been evaluated for detecting *Salmonella Typhi* rather than NTS [136].

Antibody-based tests lack specificity and cannot distinguish between invasive and gastrointestinal diseases. They have high false-positive rates in endemic populations, making them unreliable and not recommended for use [4,137].

PCR is a molecular technique that identifies specific DNA sequences quickly and with high sensitivity. It can be automated and test for multiple targets simultaneously [138]. In current practice, a well-validated multiplex PCR method is used to target multiple enteric pathogens. This method can identify *Salmonella* in stool samples by detecting the *ttr* and *invA* genes [139]. However, another study describes a monoplex PCR assay that detects the same *Salmonella* genes but includes a selenite pre-enrichment step. This method showed increased sensitivity compared to the validated multiplex PCR, likely due to using selenite pre-cultured stool instead of extracting DNA from raw stool samples. The monoplex test is also more cost-effective and faster than the multiplex method, even with the selenite enrichment step [138]. Nonetheless, rapid molecular methods should be used cautiously. A recent study found that using these techniques for diagnosing *Salmonella* gastroenteritis led to higher rates of hospital admissions, obtaining blood cultures, and antibiotic usage. This indicates a potential tendency towards overmedicalization of uncomplicated cases of *Salmonella* gastroenteritis, prompting a change in clinical practices. Rapid molecular methods offer little benefit for uncomplicated illnesses and should be reserved for complicated or invasive cases [140].

Loop-mediated isothermal amplification (LAMP) is a molecular technique used to amplify nucleic acids. It employs a specific set of four or six primers that bind to six or eight distinct regions on the target gene, ensuring high specificity. Reactions occur at a constant temperature, typically between 60 °C and 65 °C. Unlike traditional methods, post-amplification electrophoresis is unnecessary; detection can be achieved through visual observation without specialized equipment. LAMP technology offers a sensitivity that is one to two orders of magnitude higher than conventional PCR techniques [141]. It has been proposed for use as a point-of-care (POC) diagnostic method, allowing testing near the patient in settings with limited laboratory infrastructure. Given the ongoing challenge of timely analysis of blood samples for iNTS infection, particularly in disadvantaged regions with high incidence rates, LAMP technology could provide a practical solution [142].

With the emergence of next-generation sequencing (NGS) techniques, genomic typing tools have gained increased popularity and effectiveness. These methods are predominantly utilized for serotyping, aiming to replace traditional serotyping approaches [143]. They are superior to the actual gold standard for subtyping, as PFGE is unable to separate very closely related strains [144]. In recent years, there has been a significant decrease in the cost of genome sequencing, making this technology increasingly accessible worldwide. This technology is widely regarded as the most advanced technique for identifying infection clusters and is strongly recommended by international authorities [145]. However, for effective real-time surveillance using genome sequencing, it is essential to develop and implement a comprehensive cross-sectional strategy that encompasses public health, veterinary, food, feed, and environmental sectors. Many countries have established or are in the process of establishing national genome sequencing-based surveillance systems and platforms, such as Public Health England in the United Kingdom and Switzerland. Simultaneously, large international platforms like Pathogenwatch, INNUENDO NCBI pathogen detection, and GenomeTrakr have been developed to facilitate the analysis of multinational genome sequence data for the surveillance and investigation of cross-border transmissions and outbreaks [146]. Certainly, as time progresses, technologies using genome sequencing are likely to become the gold standard, replacing the current methods.

In summary, while rapid molecular techniques such as PCR offer promising advancements in diagnosing NTS infections, exercising caution is crucial due to their potential to overmedicalize uncomplicated cases. Their application should be reserved for complicated or invasive diseases. Achieving a balance between innovation and prudent clinical practice is imperative for enhancing patient care in the diagnosis of NTS.

## 5. Treatment of NTS Infection

### 5.1. Current Disease Management

**Figure 4:**
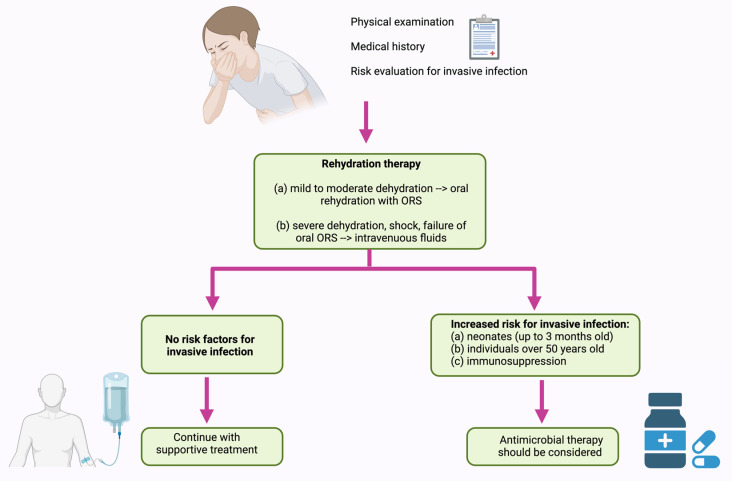
Management of NTS infection. Image created with BioRender.com. ORS: oral rehydration solutions.

In 2017, the Infectious Diseases Society of America (IDSA) elaborated an evidence-based guideline for managing infants, children, adolescents, and adults in the United States with acute or persistent infectious diarrhea (Figure 4). According to this guideline, in NTS infection the first line of therapy should be supportive treatment, specifically rehydration therapy [147].

As such, reduced osmolarity oral rehydration solutions (ORS) are recommended for mild to moderate dehydration in infants, children, and adults with acute diarrhea, as well as in individuals experiencing mild to moderate dehydration associated with vomiting or severe diarrhea. Nasogastric administration of ORS may be considered when oral intake cannot be tolerated. Isotonic intravenous fluids (lactated Ringer’s or normal saline solution) should be administered in cases of severe dehydration, shock, altered mental status, failure of ORS therapy, or ileus. Antimicrobial therapy is not recommended for treating uncomplicated *Salmonella* gastroenteritis. This approach may prolong asymptomatic *Salmonella* carriage and disrupt the microbiome, without shortening the duration of the illness, diarrhea, or fever [147].

Antimicrobial therapy should be considered for individuals at increased risk for invasive infection. This group includes neonates (up to 3 months old), individuals over 50 years old with suspected atherosclerosis, and those with immunosuppression, cardiac disease (valvular or endovascular), or significant joint disease. If the pathogen is susceptible, treatment options include ceftriaxone, ciprofloxacin, trimethoprim-sulfamethoxazole, or amoxicillin [147]. The European Society for Pediatric Gastroenterology, Hepatology, and Nutrition (ESPGHAN) and the European Society for Pediatric Infectious Diseases (ESPID) offer the same recommendations for managing acute gastroenteritis in Europe [148]. China adheres to the same recommendations, suggesting a lactose-free diet during the infectious episode, as it can shorten the duration of diarrhea, along with zinc supplements [149].

### 5.2. Antimicrobial Resistance (AMR)

Health authorities are increasingly concerned about rising cases of gastroenteritis and sepsis caused by NTS strains that are resistant, or even multi-resistant, to common antimicrobials like beta-lactams, aminoglycosides, and quinolones. AMR in NTS has arisen from the extensive use of antibiotics in food animal production and the unrestricted use of antibiotics in clinical settings [24]. Bacteria acquire antimicrobial resistance through horizontal gene transfer (HGT), which can happen via three main mechanisms: transformation, transduction, or conjugation. The gut microbiota exhibits a higher rate of HGT compared to bacteria in other environments. Although contact-dependent conjugation between microbiota members like *Enterobacteriaceae* is restricted by commensal bacteria, the inflammatory response to pathogens can boost the occurrence of conjugative HGT. Specifically, infections involving NTS can promote the thriving of *Enterobacteriaceae*, thereby increasing HGT between NTS and commensal microbes [115].

The latest AMR report from the EFSA and the European Centre for Disease Prevention and Control (ECDC) highlights findings from coordinated AMR monitoring conducted between 2020 and 2021 [6]. In 2021, in Europe, NTS isolates from humans showed notable increases in resistance to ampicillin (25.2%), sulfonamides (25.6%), and tetracyclines (25.1%) [6]. However, significant variations exist across continents (Table 1).

For instance, the USA reported lower rates of ampicillin resistance compared to Europe (6.6%) [24], while China reported a much higher resistance rate of 73.4% [150]. Resistance to ampicillin in Salmonella typically involves the production of β-lactamases [153].

In Europe, resistance to third-generation cephalosporins among human isolates remained low in 2021, with rates of 1.1% for ceftazidime and 1.1% for cefotaxime [6]. In the USA, resistance rates were comparable, with 3% resistance to ceftriaxone [24]. However, in China, nearly 20% of human NTS isolates from diarrhea cases showed resistance to third-generation cephalosporins, significantly higher than in the USA and Europe. Despite this, there was no observed increase in resistance trends, indicating that these antimicrobials remain effective for most NTS infections [150]. Resistance to ceftriaxone is primarily mediated by genes encoding extended-spectrum β-lactamases (ESBLs) such as *bla*_TEM_, *bla*_SHV_, *bla*_CMY_, *bla*_CTX-M_, and *bla*_OXA_ [154].

Fluoroquinolone resistance in Europe, especially to ciprofloxacin, averaged 14.9% across various NTS strains [6]. In the USA, fluoroquinolone resistance is notably lower compared to Europe, at 3% [24], while China reports similar rates to Europe, around 16.2% [150]. Resistance to ciprofloxacin primarily arises from dual mutations in the *gyrA* gene and a single mutation in the *parC* gene, with mutations in *gyrB* and *parE* genes being less common. Additionally, plasmid-mediated quinolone resistance (PMQR) and efflux pumps contribute to the development of low-level resistance to quinolones and fluoroquinolones [154].

Carbapenemase-producing *Salmonella* spp. were absent in human cases throughout Europe in 2021 and were also not found in animal isolates from 2020 to 2021 [6]. However, research by Ping-Chun Hsu et al. suggests that *Salmonella* strains producing ESBL and AmpC β-lactamases can develop carbapenem resistance through the loss of two specific porins (OmpC_378 and OmpD). They found that most *Salmonella* serovars possess seven porin paralogs, and the absence of OmpC_378 and OmpD is critical for carbapenem resistance development [155].

High rates of MDR were observed among *Salmonella* spp. reported in human cases in Europe, totaling 22.6% [6]. In USA the MDR rate is much lower than in Europe (10.3%) [24]. A particular situation is in China, where the incidence of MDR notably surpasses those from other countries, ranging from 40% to 81% [151,152].

### 5.3. Alternative Therapeutics

#### 5.3.1. Probiotics

Given the increasing occurrence of severe cases of NTS infection due to the rise in AMR, new therapeutic alternatives are needed. Thus, attention has been directed towards the use of probiotics for the treatment of gastroenteritis. The term of probiotic is defined by WHO as “living microorganisms that, when administered in adequate amounts, confer benefits to the health of the host” [156]. Probiotics enhance intestinal health by adhering to host cells, reinforcing the mucosal barrier, and competing for fermentation substrates to prevent pathogen adherence [157]. They also support gut health by promoting the growth of beneficial bacteria, blocking pathogen access to the gut, reducing inflammation, and optimizing nutrient absorption. Probiotics exert immunomodulatory effects through interactions with various immune cells, stimulating mucosal immunity through immunoglobulin A release and enhancing defense against pathogens via cytokine expression like IL-6, IL-10, and TNF-β [158].

Studies on the prophylactic and therapeutic effects of probiotics have shown antagonistic properties against NTS, with most research conducted on animals [159,160,161,162,163]. Due to the relative novelty of probiotics, human trials are limited, especially those evaluating the efficacy of probiotics against specific pathogens. Most human studies focus on infectious diarrhea and rarely target individual etiological agents. That is why the ESPGHAN Special Interest Group on Gut Microbiota and Modifications provides updated guidelines on using probiotics for specific pediatric gastrointestinal disorders, responding to the growing demand for alternative therapies in acute gastroenteritis [164]. Based on systematic reviews and meta-analyses from 2010 to 2020, the Work Group did not find sufficient high-quality RCTs for any strain showing clear benefits in treating acute gastroenteritis. Therefore, their recommendations are weak (conditional). Consequently, they recommended the following strains in descending order based on trial evaluations: *Saccharomyces boulardii*, *Lactobacillus rhamnosus* GG, *Limosilactobacillus reuteri* (formerly *Lactobacillus reuteri*), *Lactobacillus rhamnosus* 19070-2, and *Lactobacillus reuteri* DSM 12246. Conversely, the Work Group strongly advised against using *Lactobacillus helveticus* R0052 and *Lactobacillus rhamnosus* R0011, and weakly advised against *Bacillus clausii* strains O/C, SIN, N/R, and T due to their lack of efficacy in symptom improvement [164]. Moreover, a study published in 2023 documented an infant who developed sepsis from *Bacillus clausii* after receiving a probiotic containing this bacterium as an initial treatment for acute diarrhea [165].

#### 5.3.2. Vaccination

Given the increasing number of cases of NTS infection, as well as the rise in cases of invasive infection [30], along with the increasing antibiotic resistance rate and the possibility of developing significant complications, the development of an effective vaccine would be a valuable tool in reducing morbidity and mortality. To date, there is no commercially available vaccine against NTS. Although an established immune marker for protection against iNTS has not been confirmed, defense is likely mediated through both T cell-dependent and -independent pathways. Antibodies contribute to protection by directly killing bacteria through complement activation and by enhancing oxidative burst-mediated killing and phagocytosis via opsonization [166,167]. There are several vaccines under study being developed for protection against NTS, which we will briefly introduce next.

The NTS-GMMA vaccine, utilizing GMMA technology, is a bivalent vaccine designed to protect against iNTS, combining modified *S.* Typhimurium and *S*. Enteritidis strains. GMMA, or “Generalized Modules for Membrane Antigens”, involves using bacterial outer membrane vesicles to stimulate immune responses [168]. Pre-clinical trials of iNTS-GMMA in mice showed promising results, inducing strong antibody responses against *S.* Typhimurium and *S.* Enteritidis O-antigens, along with potent bactericidal activity in vitro [169,170]. Phase 1 clinical trials are currently ongoing at the University of Oxford, marking the first investigation of the iNTS-GMMA vaccine in humans [171].

Live attenuated bacterial vaccines are extensively researched for their potential advantages. Numerous preclinical studies have evaluated potential live-attenuated iNTS vaccine candidates [172,173]. One new candidate, a double D-glutamate plus D-alanine auxotroph of *S.* Typhimurium, showed promising results in mice when administered orally [174]. However, only two clinical studies in humans have been conducted so far, which were discontinued due to a weak response [175,176].

The *Salmonella* glycoconjugate approach targets protective antibody responses against surface polysaccharides, including core and O-polysaccharides (COPS) [172]. Recently, a glycoconjugate vaccine combining *S*. Typhimurium O-specific polysaccharide with recombinant T2544 provided protection against *S.* Typhi, *S*. Paratyphi, *S.* Typhimurium, and cross-protection against *S*. Enteritidis in mice [177]. Researchers explored flagellin’s potential to induce protective immunity, finding that immunizing mice with flagellin alone or in combination with O-antigen stimulates protective responses [178]. Baliban et al. presented the preclinical development of *S.* Enteritidis and *S.* Typhimurium COPS:FliC conjugates, demonstrating high immunogenicity in adult and infant mice and protection against lethal systemic challenge [179]. Bharat Biotech has licensed COPS:FliC technology, planning the first human clinical trial for a trivalent typhoid-iNTS conjugate vaccine [172].

The Multiple Antigen Presenting System (MAPS) uses biotin-rhizavidin affinity to create polysaccharide-protein complexes, offering a promising alternative to conventional conjugation methods [180]. A recent study details the preclinical development of the first MAPS vaccine candidate against NTS, featuring Vi from *S.* Typhi, O-specific Polysaccharide (OSP) from *S.* Paratyphi A, *S*. Enteritidis, and *S.* Typhimurium, along with *Salmonella*-specific protein SseB. This vaccine demonstrated strong antibody responses to each component [181].

The current vaccine development process for NTS appears to be lacking, which reflects a deficiency in both interest and resources to propel clinical testing, rather than indicating the infeasibility of developing an NTS vaccine. Such a vaccine would be advantageous not only for travelers from developed countries but also as an early childhood vaccine in endemic regions. Additionally, it would help prevent invasive disease and significant complications in high-risk populations.

## 6. Conclusions and Future Perspectives

Despite the coordinated efforts of public health programs aimed at controlling and preventing it, NTS infection remains a substantial public health issue. NTS infection presents significant risks due to its high rates of morbidity and mortality, as well as the potential for patients to develop significant short- and long-term complications. Clinicians should be mindful of the potential complications of *Salmonella* infection in patients experiencing an unfavorable clinical course.

While NTS infection is a notable public health concern, its pathogenesis remains incompletely understood. Enhanced understanding of its pathogenesis could offer opportunities for developing novel therapeutic targets. Furthermore, gaining insight into the mechanisms that lead to invasive infections could aid in developing preventive therapeutic strategies, which would be particularly advantageous for populations at risk of developing iNTS infection.

Health authorities are increasingly alarmed by the growing incidence of gastroenteritis and sepsis caused by NTS strains that exhibit resistance, including multidrug resistance, against common antimicrobials such as beta-lactams, aminoglycosides, and quinolones. The emergence of antimicrobial resistance in NTS is linked to widespread antibiotic use in food animal production and unrestricted antibiotic usage in clinical settings. With the increase in antimicrobial resistance rates, there is a pressing need for new therapeutic options. Probiotics have been considered as one such alternative; however, studies on their prophylactic and therapeutic effects have revealed conflicting results when used against NTS. Therefore, there is a growing need for further research to explore the interactions between NTS and various probiotics, assessing their potential benefits in treating NTS infections. Another therapeutic alternative would be the development of an effective vaccine, which would serve as a valuable tool in reducing morbidity and mortality. Currently, there is no commercially available vaccine against NTS. Several vaccines are under study for protection against NTS, with Phase 1 clinical trials currently underway at the University of Oxford, marking the initial investigation of the iNTS-GMMA vaccine in humans. Since the development of an NTS vaccine appears feasible, greater efforts should be directed towards this goal. This initiative stands to benefit not only travelers from developed countries but also children in endemic regions and high-risk populations.

## Figures and Tables

**Table 1:** Antimicrobial resistance profiles of NTS across the world.

Antibiotic	Europe	USA	China	Molecular Mechanism of Resistance	Year of Antimicrobial Resistance Assessment
Ampicilin	25.2%	6.6%	73.4%	β-lactamases	Europe 2020–2021 [6]
					USA 2004–2016 [24]
					China 2014–2021 [150]
Third-generation cephalosporin	1.1%	3%	20%	genes encoding ESBL *bla*_TEM_ *bla*_SHV_ *bla*_CMY_ *bla*_CTX-M_ *bla*_OXA_	Europe 2020–2021 [6] USA 2004–2016 [24] China 2014–2021 [150]
Fluroquinolones	14.9%	3%	16.2%	dual mutations in the *gyrA* gene single mutation in the *parC* gene rarely mutations in the *gyrB* and *parE* genes PMQR ^1^ efflux pumps	Europe 2020–2021 [6] USA 2004–2016 [24] China 2014–2021 [150]
MDR	22.6%	10.3%	40–81%		Europe 2020–2021 [6]
					USA 2004–2016 [24]
					China 2012–2019 [151,152]

^1^ plasmid-mediated quinolone resistance; MDR: multidrug resistance.
